# Interventions to suppress the coronavirus pandemic will increase unemployment and lead to many premature deaths

**DOI:** 10.1177/1403494820947974

**Published:** 2020-08-25

**Authors:** Måns Rosén, Magnus Stenbeck

**Affiliations:** 1 Karolinska Institutet, Sweden; 2 Karolinska Institutet, Institute of Environmental Medicine, Sweden

**Keywords:** Coronavirus, COVID-19, interventions, unemployment, mortality, economic recession

## Abstract

**Background and aims:**

Interventions to suppress the coronavirus pandemic have led to economic recession and higher unemployment, which will increase mortality and decrease quality of life. The aim of this article is to estimate the consequences on mortality and life expectancy of increased unemployment rates due to the coronavirus pandemic in Sweden and other countries.

**Methods:**

Based on recent increases and expected future unemployment rates due to the coronavirus pandemic, results from a systematic review and data from vital statistics in Sweden, the number of premature deaths due to unemployment in Sweden have been estimated.

**Results:**

Based on our assumptions, the calculations show that if the number of unemployed persons in Sweden increases by 100,000, one may expect some 1800 more premature deaths during the following 9 years. If the duration of the recession is limited to 4 years, excess deaths due to unemployment may be around 800. On average, the unemployed will lose 2 years of their remaining life expectancy. In many other countries unemployment rates have or are estimated to rise more than in Sweden, sometimes two- or threefold, suggesting hundreds of thousands of excess deaths due to unemployment.

**Conclusions:**

**Interventions to suppress the coronavirus pandemic include the shut-down of economic activities and lead to increased all-cause mortality. These public health effects must be considered in the decision-making process and should be added to overall estimates of the effects of the pandemic on public health.**

## Background

Before political decisions are taken, the benefits of an intervention must be weighed against the risks and the costs. In this time of coronavirus (corona) pandemic, leaders around the world are taking actions on a day-to-day basis to stop the immediate dispersion of the infection. This is done to diminish or slow down the pandemic and is often based on epidemiological simulation models [1,2] estimating large numbers of COVID-19 related deaths if actions are not taken. Such models do not consider that many of the interventions creating economic crisis and unemployment have direct effects on public health. Closing boundaries, schools, shops or restaurants and other interventions have already created enormous economic problems for the transport sector, industry and not least for small businesses such as restaurants and hotels. Closing preschools and schools will, according to the Public Health Agency of Sweden, increase health personnel absence from work by 25% [3] A recent study shows that school closure for COVID-19 is a questionable public health measure because many in the healthcare workforce will have to stay at home [4] Economists and finance ministers around the world now predict that unemployment will raise substantially. Gross national product will decrease. The length of this recession period will decide the impact on public health. Many studies show that extended unemployment will increase the risk of premature deaths [5–9], increased hospitalization [10] and decreased quality of life [11].

The Swedish Institute of Economic Research forecasts an unemployment rate of 11% in 2021, which is an increase of about 60% [12] In Norway, the unemployment rate more than tripled to 10.7% by the end of March 2020 [13] The unemployment rate of the UK in the first quarter of 2020 was 3.67%, but it is estimated to reach about 10% in the second quarter of 2020 [14] In the USA, the unemployment rate skyrocketed to 14.7% in April 2020. Compared to the first quarter of 2020, the unemployment rate has increased nearly four times in the US [15]

There are also other negative consequences of corona interventions. In the short run, planned cancer surgery and other important operations were postponed to create resources for the intensive care of COVID-19 patients. Postponed treatment is probably of less overall importance, but poverty, increased number of crimes and less resources for healthcare due to decreases in gross national products are more serious problems. This is especially true in developing countries with already limited economic resources and poorer health.

The aim of this article is to estimate the public health consequences on mortality and life expectancy of increased unemployment rates due to the corona pandemic in Sweden and other countries.

## Methods

The basis for our calculations is a previously published systematic review and meta-analysis of the association between unemployment and all-cause mortality [7]. The review included 42 studies showing relative hazard ratios of between 1.25 and 1.77 for unemployed people compared to the working population. The effects were shown to depend on the age of the unemployed persons.

The exposure to unemployment was classified based on single measurements of current labour market status without regard to the length of unemployment or employment. Hence, the effect of the duration of unemployment was not estimated in the systematic review. The average follow-up period was 9 years. The risks were highest during the first part of that period but elevated for the full 9 years. The estimates presented in the review and used in our computations are average excess risks for the entire 9-year period. Assuming the current recession will be of limited duration, we made the conservative assumption that the number of premature deaths due to unemployment may last for only 4 years.

We used two outcome measures:

1. the number of excess premature deaths due to unemployment and
2. the loss of remaining life expectancy among the unemployed.

We used census and official labour market survey information on the population of Sweden in 2019. The normal age span of official unemployment statistics is 16–74 years, but because the labour market participation of the youngest and oldest ages is very limited, we used information on the population aged 20–64 years [16] The overall unemployment rate in this group is slightly lower than that for the entire age span (in April 2019 approximately one percentage point). The baseline risks of death in the population were estimated for each sex and age. Baseline risks varied between 0.02% for 20-year-old females to 0.82% for 64-year-old males. The risks for unemployed were estimated by multiplying the baseline risks by the relative hazard ratios reported from the systematic review in age classes: 1.73 for ages 20–39, 1.77 for 40–49 and 1.25 for 50–64. This yielded a rise in the mortality risk for the unemployed by between 0.02 and 0.24 percentage points up to a maximum annual death risk of 1.02% for 64-year-old males. We used the same hazard ratios for both sexes because we found no sex- and age-specific estimated hazard ratios in the systematic review.

The overall rate of unemployment at baseline was 5.6% in the population 20–64 years of age [17]. We used the rates of employed and unemployed for each sex and age classes 20–24, 25–34, 35–44, 45–54 and 55–64 as the baseline. We increased the proportions of unemployed people by assuming an additional unemployment of 100,000 persons (50,000 of each sex), which represents some 34% for males and 36% for females. In the future, this number can easily be adjusted to the actual figures of additional unemployment. As a comparison, during the financial crisis between 2008 and 2010, unemployment in Sweden rose by some 35% in the first year and close to 40% over 2 years [18] This represents a moderate increase compared to the forecast of 60% made by the Swedish Institute of Economic Research cited above.

The expected number of deaths among the assumed number of new unemployed people was subtracted from the expected number of deaths in the general population for each sex and age to arrive at estimates of the increased number of deaths in 1 year. According to the systematic review, the enhanced death risks prevailed during the entire 9-year period. We therefore multiplied the aggregate number of expected excess deaths by 9 to achieve a total estimate of excess deaths.

The expected loss of remaining years of life for an individual of age i=20,..,64 was computed for each sex and age using conventional life table methodology [19]

Finally, our computations were used to illustrate possible effects in other countries. Based on the Swedish calculations [16], considering the size of the country-specific labour forces [20] and unemployment rates of January 2020 [21], we estimated the excess mortality in some other countries using the scenarios that unemployment rates would increase by 50%, 100%, 200% and 300% respectively.

## Results

According to official statistics for April 2019 some 370,000 people were unemployed in Sweden. Of these, almost 286,000 were between 20 and 64 years old. By assuming an increase in unemployment by 100,000 (35%) people, there would be an annual excess number of premature deaths due to unemployment of 200 per year, 1800 after 9 years of follow-up (Table I), or 800 after 4 years of follow-up.

**Table I.** Estimated number of excess deaths and life years lost per 100,000 unemployed persons per year and after 4 and 9 years of follow-up in 2020 in Sweden.

Increased number of unemployed persons in Sweden 2020 compared to 2019	Annual excess deaths	Excess deaths after 9 years of follow-up	Excess deaths after 4 years of follow-up	Additional years of life lost after 4 years of follow-up
Men + 50 000	126	1134	504	1058
Women +50 000	74	666	296	592
Total +100 000	200	1800	800	699

The estimated number of deaths is based on hazard ratios of unemployed persons at study start and followed up for 9 years from the systematic review (6). Years of life lost are based on our calculations that on average unemployed men lost 2.1 years and women 2.0 years compared to employed people in the same age group.

We also calculated the loss of remaining life expectancy among unemployed people. In general, men would lose 2.1 years and women 2.0 years.

All countries differ in their economic and health situations as well as in how well health services and social security systems function. It is therefore difficult to estimate the number of premature deaths in different countries directly based on our national data. In spite of this and to illustrate the magnitude of the problem in different countries, we applied Swedish estimates to the labour forces in some other countries to estimate the public health consequences of unemployment (Table II). At present, we do not know how long the economic recession will last. If it is more restricted in time, we could anticipate somewhat less severe consequences. Using a conservative approach, we therefore restricted the negative effects to only 4 years.

**Table II.** Estimated number of deaths due to increase in unemployment rate after 4 years of follow-up in different countries according to their labour force. The estimation is based on a systematic review (6) applied to Swedish population vital statistics.

Country	Labour force	Unemployment rate M1, 2020	Unemployment increases by 50%	100%	200%	300%
Sweden	5,361,000	7.2	1148	2295	4592	6887
Denmark	2,998,000	4.9	642	1283	2 568	3851
Norway	2,797,000	3.7	599	1197	2 396	3593
Spain	22,750,000	13.9	4872	9739	19 487	29,226
Italy	25,940,000	9.4	5555	11,105	22 219	33,324
France	30,680,000	8.0	6570	13,134	26 279	39,413
UK	33,500,000	3.8	7174	14,341	28 695	43,036
USA	160,400,000	3.6	34,348	68,666	137 392	206,058

Labour force: CIA. The World Factbook: https://www.cia.gov/library/publications/the-world-factbook/rankorder/2095rank.html

Unemployment rate, January 2020 [20]. OECD (2020), Unemployment rate (indicator). doi: 10.1787/52570002-en (accessed 1 July 2020) [21].

The predicted increase in unemployment suggest an 100–300% increase in Norway [13], nearly 200% in the UK [14] and more than 300% in the USA [15] A 100% increase in Norway would then imply about 1197 deaths, a 200% increase in the UK would imply 28,695 deaths and a 300% increase in the USA would imply 206,055 deaths (Table II).

## Discussion

Based on our assumptions, the calculations show that if the number of unemployed persons in Sweden increases by 100,000 there would probably be some 1800 additional premature deaths for people aged 20–64. On average, the unemployed will lose about 2 years of their remaining life expectancy. The unemployed are on average much younger than those dying from corona. According to Statistics Sweden, a Swedish citizen at the age of 45 has an average of 38 remaining years. At the age of 80 they have 9 remaining years and 70% of those dying from corona are over 80 years of age. The total number of life years lost for the unemployed will therefore be substantial, as well as the loss of quality adjusted life years.

There are several limitations with these calculations. Firstly, as mentioned in the methods section, there was no way to assess the impact of the duration of unemployment at the individual level. In most studies, unemployment was only measured once in the beginning of the follow-up period. Some of the participants had been unemployed for a short time and others were unemployed for longer. Hence, the follow-up started with mixed groups in terms of the history of unemployment. The systematic review reported a higher excess risk during the first part of the follow-up than later on. This may be due to that a greater share of the unemployed had returned to work toward the end of the follow-up period.

Secondly, on the aggregate level we do not know the duration of the wave of excess unemployment. If the recession period is short, the negative consequences will be less severe. Our estimated 9 years of follow-up may be somewhat exaggerated. We have therefore added a more conservative approach where we assume excess deaths will end after 4 years. Among the 42 studies included in the systematic review, those with shorter follow-ups of 3–5 years have similar hazard ratios to those with longer follow-ups.

Thirdly, excess risks for the unemployed in the systematic review are based on studies from different parts of the world with different unemployment rates, social security systems and country-specific economic stability. Finally, mortality will probably be affected by whether we are in a recession or an economic boom. Unemployed people during a recession or an economic boom will be quite different sectors of the population in terms of their basic health status when they become unemployed. A study after the 1992–1996 Swedish recession showed excess all-cause mortality, but somewhat lower hazard ratios than in the systematic review we used for our calculations [9] A follow-up study showed the duration of unemployment was especially positively related to alcohol and external causes of death in a dose-response manner [22]

Nevertheless, the potential negative effects of interventions to suppress the spread of corona are far from neglectable and should be taken into consideration when decision-makers take action. Can we find interventions that have less severe consequences for the economy and unemployment rates? How much of a lockdown of normal economic activity is acceptable? Predictions indicate the unemployment rate will increase less in Sweden than in countries such as Norway, the UK and especially the USA. As an example, we estimated that an increase in the unemployment rate of 100% or 300% in Norway would mean 1197 and 3593 additional premature deaths respectively whereas 233 COVID-19 related deaths were reported by 26 May 2020. In contrast, Norway has a developed social insurance system and a sound economic basis. Hence, economic interventions may be able to reduce substantially premature deaths due to unemployment. At this stage of the pandemic, it is too early to evaluate the effects and the trade-off between COVID-19-related deaths and deaths due to unemployment

Important questions not raised in this paper include how a decrease in gross national product will influence future healthcare resources and the impact patient outcomes. Postponed treatments may also have negative effects, as well as additional factors such as alcohol abuse and mental health problems unrelated to unemployment. In contrast, economic lockdown may have positive effects on air pollution and may lead to fewer work-related accidents etc.

Our calculations on premature deaths caused by unemployment between different countries (Table II) are somewhat speculative and very tentative, but we think it is a way of creating international awareness of the overall consequences of corona interventions. A report from CNBC in US reported federal estimates of 47 million job losses in the US, leading to an unemployment rate of 32% [23], a predicted increase by over 900% compared to the February unemployment rate of 3.5% [24] This seems to be an incredibly high prediction, which of course also would mean a much more severe increase in all-cause mortality of several hundreds of thousands of lives in the USA only.

Economic recession increases unemployment, but it also increases the number of people living in poverty for several other reasons. Together with King’s College London and the Australian National University, Oxfam has estimated that an additional 434 million people in the world could become poor because of the corona pandemic [25] Besides a poor quality of life, poor people have substantially lower life expectancy and many low-income countries may also have difficulties during the pandemic in accessing medicines and medical equipment used to treat other public health problems. However, this paper has focused on the expected effects in high-income countries.

So far, Sweden has had quite high death rates of per 1 million inhabitants among those with corona. In contrast, Swedish corona protection policies have partly been apprehended and less restrictive, which may limit disturbances to normal economic activity. Economic analyses so far indicate that unemployment rates have increased somewhat less in Sweden than some other countries [12–15] Whether this has been successful remains to be seen. Nevertheless, increased mortality following rising unemployment must be included in the overall assessment of the long-term effects of the pandemic.

Countries with good economy and well-functioning social security systems for the entire population will probably manage the recession better than developing countries with already constrained economies and less-developed healthcare systems.

It is evident that the number of deaths attributable to economic recession and unemployment will be quite high and must be taken into account when the consequences of the pandemic are summarized. In retrospect, it will be possible to evaluate the long-term impact on the mortality from all causes to get an estimate of the overall damage caused by the epidemic. Our main message is that we have to weigh up the public health benefits against the public health risks of intervening to suppress corona. More international collaboration is needed to limit the negative consequences of the interventions on trade and business.
